# Population-based survey regarding factors contributing to expectation for death at home

**DOI:** 10.1186/s12930-018-0044-z

**Published:** 2018-07-11

**Authors:** Tomoya Tsuchida, Hirotaka Onishi, Yoshifumi Ono, Ako Machino, Fumiko Inoue, Manabu Kamegai

**Affiliations:** 1 Division of General Practice, Department of Internal Medicine, Kawasaki Municipal Tama Hospital, 1-30-37 Shukugawara, Tama-ku, Kasawasaki-shi, Kanagawa-ken 214-8525 Japan; 2 International Research Center for Medical Education, Graduate School of Medicine, Faculty of Medicine, The University of Tokyo, Central Building 7-3-1 Hongo, Bunkyo-ku, Tokyo, 113-0033 Japan; 3 Division of Pediatrics, Kawasaki Municipal Tama Hospital, 1-30-37 Shukugawara, Tama-ku, Kasawasaki-shi, Kanagawa-ken 214-8525 Japan; 4 Department of Nursing, Kawasaki Municipal Tama Hospital, 1-30-37 Shukugawara, Tama-ku, Kasawasaki-shi, Kanagawa-ken 214-8525 Japan; 5 Ai Clinic Nakazawa Yuimaru Nakazawa A-1, Nakazawa, Tama-shi, Tokyo 206-0036 Japan

**Keywords:** Terminal care, Surveys and questionnaires, Multivariable analyses

## Abstract

**Background:**

In 2015 in Japan 12.7% of people die at home. Since the government has no policy to increase the number of hospital beds, at-home deaths should inevitably increase in the near future. Previous researches regarding expected place of death have focused on end-of-life patients. The aim of this study is to clarify the percentage and factors of senior people who expect at-home deaths whether they are end-of-life or not.

**Methods:**

Using cross-sectional questionnaire survey data which had been taken by a research group with the support from Tama City Medical Association (Tokyo) in 2014, univariable and multivariable logistic regression analyses were conducted to identify associations among factors. The dependent variable was the expected site of death and other factors were set as independent variables.

**Results:**

Of 1781 respondents, 46.5% expected at-home deaths. Data from 1133 people were analyzed and 46.5% of those wanted at-home deaths. Factors significantly associated with expectation of at-home death were men, stand-alone houses for dwelling, expectation to continue life in Tama city, twosome life with the spouse, healthiness, and economic challenge.

**Conclusion:**

Percentage of those who expected at-home deaths was much higher than the latest percentage of at-home deaths. Some factors associated with expectation of at-home deaths in this study have never been discussed.

## Background

**Fig. 1:**
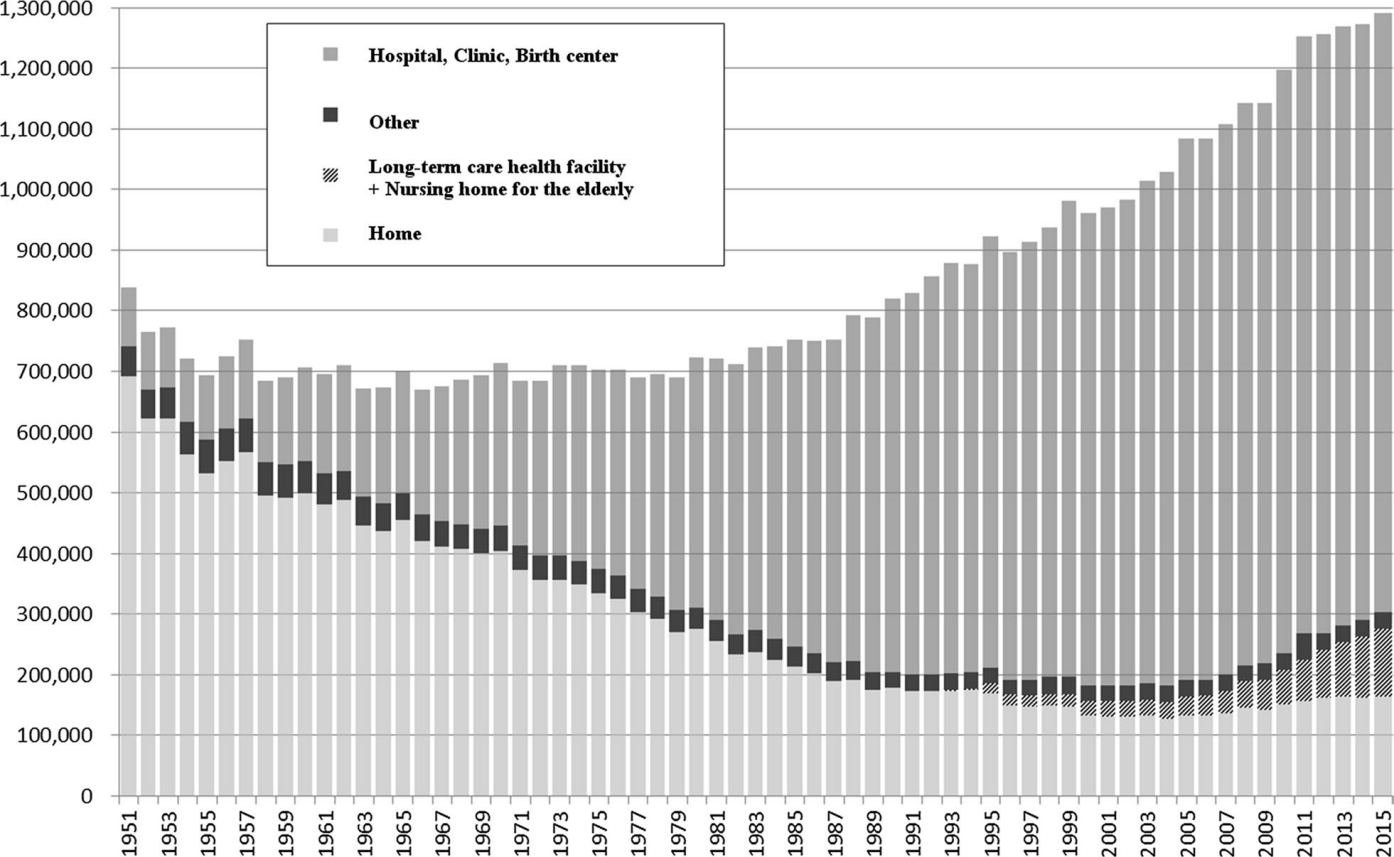
Trends in deaths by place of death. Deaths at long-term care health facilities before 1989 and those at nursing homes for the elderly before 1995 are included in the category of home deaths. Excerpt from Trends in deaths by place of death. Annual Vital Statistics Report (Final) 2015, Ministry of Health, Labor and Welfare

As Japan becomes an aging society with declining birth rates and increasing mortality rates, the gap between preferred and actual place of death is currently an important issue in medical care and welfare. It is estimated that the number of annual deaths in Japan will increase from 1.29 million in 2015 [1] to 1.67 million in 2040 [2]. In the 1950s, homes accounted for 82.5% of places of death. In 2015, homes accounted for only 12.7% of places of death while hospitals accounted for 78.4%. On the other hand, the idea of ending life in a familiar place has become common in the last decade. Home deaths increased slightly from 12.2% in 2005 to 12.7% in 2015. The death rates for those who died at long-term care health facilities or nursing homes for the elderly increased from 2.8% in 2005 to 8.6% in 2015, showing a considerable increase [1] (Fig. 1). In addition, according to the Working Group for the Analysis and Discussion of Medical and Nursing Care Information, in the Expert Panels on the Promotion of Reforms with Medical and Nursing Care Information of the National Council on Social Security System Reform, hospital bed capacity in 2013 was 1.35 million, and the estimated hospital bed capacity by medical care function in 2025 is predicted to be 1.15–1.19 million [3]. There is no plan to increase hospital bed capacity in the future. It can be inferred that more people will die at home or in care facilities if there is no reduction in hospital stay, among other aspects. Thus, it is important to explore factors that affect place of death in predicting the trend of medical and nursing care.

A systematic review of factors influencing death at home in terminally ill patients with cancer identified the following items as factors strongly associated with home death: patients’ low functional status, patients’ preferences, use and intensity of home care, living arrangements, and extended family support [4]. In addition, meta-ethnography in Wahid et al. lists the following items as barriers to home death: lack of knowledge, skills of and support among informal carers and healthcare professionals, informal carer and family burden, recognizing death, inadequacy of processes such as advance care planning and discharge, and inherent patient difficulties due to medical conditions or social circumstances. It also lists the following items as factors promoting home death: support for patients and healthcare professionals, skilled staff, coordination and effective communication [5].

Regarding the situation in Japan, the results of “the Surveys of Palliative Care” 1998 by “the Palliative Care Meeting” showed that those who wanted to be hospitalized in a medical institution or palliative care ward that they have visited as soon as possible accounted for 32.5%; those who wanted to receive home care and, if necessary, be hospitalized in a medical institution or palliative care ward accounted for 48.7%; and those who preferred to die at homes accounted for 9.0% [6]. These rates changed to 27.2, 52.4, and 10.9% in 2008, respectively, and the rates for those who wanted to spend a certain period of time at home or die at home slightly increased. However, this survey showed that those who wanted to die at home accounted for only 10% while many people preferred to die in a medical institution.

In a 2014 survey by the Review Committee on Awareness Surveys of Palliative Care, etc., five types of patients answered to questions about preferred place of death. Those in a coma with progressive weakness for more than half a year after a traffic accident and end-stage cancer patients who had good appetite and judgement without pain accounted for 10.3 and 71.7% of those who opted to spend their last years of life at home, respectively [7], suggesting that this type of survey shows different results depending on contexts.

The literature review by Takeu [8] identified the following items as factors commonly observed in home care patients who prefer home death: female, elderly, without pain, without breathing difficulties, bedridden, and preference for home death. It identified the following items as factors commonly observed in families and caregivers: secondary caregivers included, non-spouse or child, and preference for home death. It also identified the following items as factors commonly observed in medical care and home care services: “a visiting physician available, attending physician’s or clinic’s positive attitude toward home death, use of home care services, psychological support from the family.” Hattori et al. stated that a decrease in activities of daily living (ADL) 1 month before death is also a factor contributing to home death. They identified the following items as barriers to home death: sense of burden felt by families, anxiety at the time of sudden change, and anxiety about hospitalization at the time of sudden change [9]. Suzuki et al. listed the following items as factors enabling home death: the acceptance of the home death by the family, patient preference for home death, presence or absence of family caregivers, introduction of home care nursing, and relief of physical pain [10]. According to Sugikoto et al. in most cases, the major factor contributing to the decision on the place of death was “patients’ preferences” [11].

Tama City is located in the south-west suburb in Tokyo. It is a unique city whose population has dramatically increased within 30 years from less than 10,000 in 1960 to 140,000 in 1990 with the development of Tama New Town. Many residents are baby boomers who relocated into Tama in their thirties and forties in the late 1970s and 1980s. However, the rate of aged 65 and above in Tama City is expected to increase rapidly from 25.4% in 2015 to 32.6% in 2025 [12]. Thus, we thought it would be possible to predict the near future of Japan by analyzing the data on the preference for home death in Tama city.

The purpose of this study was to examine the characteristics of those who prefer to die at home by analyzing data from a questionnaire survey [13] in residents of Tama City aged 65 and above.

## Methods

### Purpose of the survey

The purpose of the previous survey was to build the foundation for the elderly to live peacefully in a familiar area by examining the relationship between living environment of the elderly and community. The purpose of this study was to clarify factors contributing to home death by secondary data analysis.

#### Participants

A total of 3000 people randomly extracted from 35,567 residents of Tama City aged 65 and above in September 2014 was included in this study. The questionnaires were distributed and collected by mail. The number of valid responses was 1811 (valid response rate, 60.4%).

#### Questionnaire

The questionnaire included the following items: (1) age, sex, height, and weight; (2) residential district (one district is selected from 26 districts of Tama city); (3) residence: detached houses, housing complexes of (1st or 2nd or upper floor with/without elevator); (4) year of residence; (5) preference to continue to live; (6) family type; (7) health conditions; (8) certified need for long-term care; (9) instrumental ADL (IADL); (10) contribution to community; (11) level of social activities; (12) affordability; (13) families and relatives, the frequency of communication with friends; and (14) preferred place of death (Question is “Where do you want to die? Please circle one item “medical institution, home, family’s home, facilities for the elderly, facilities covered by long-term care insurance, don’t know, others”).

#### Statistical analysis

Univariable and multivariable analyses were performed with preferred place of death as a dependent variable and other factors as independent variables. Regarding the dependent variables, preferred place of death was categorized into a dichotomous variable: “home death” (i.e., “home” or “family’s home”) and “non-home death” (i.e., “medical institutions such as hospitals,” “elderly housing with supportive services,” or “facility such as special elderly nursing home”). Responses of “do not know” and “other” are excluded from the analysis.

Changes were made to the independent variables before the analysis. Ages were categorized into three groups based on age (i.e., 65–74, 75–84, and 85+ years). Residential areas were categorized into six nominal variables by the jurisdiction of six community general support centers. Residence year was converted into a dichotomous variable of ≥ 20 years or not. Family type was categorized into four nominal variables: “living alone,” “living with only a spouse,” “living with relatives other than a spouse,” and “living with a spouse and relatives. Since independent IADL accounted for more than 80% of participants, IADL was converted into a dichotomous variable. Social activities consisted of 16 items of three-point scales. Since their Cronbach’s α was high (0.85) and factor analysis produced the first factor’s eigenvalue considerably higher than those of other factors, variables of 16 items were summarized into a continuous variable ranging from 16 to 48.

Binary logistic regression analysis was used for both univariable and multivariable analyses. For each independent variable, a crude odds ratio and an adjusted odds ratio with 95% confidence intervals (95% CI) were calculated respectively. For the multivariable analysis all the variables were entered. Data with missing values were excluded from the analysis. Multicollinearity among independent variables was defined as Pearson’s correlation coefficient > 0.9 or variance inflation factor ≥ 4. For the statistical analysis, IBM SPSS, ver. 20 (International Business Machines Corporation, Armonk, NY) was used.

#### Ethical considerations

This study was conducted as a secondary analysis of data from questionnaire survey by the Tama City Medical Association. The use of original data has been approved by the J. F. Oberlin University Research Ethics Committee. The collected data were processed as linkable anonymous data and provided by Tama City Medical Association.

## Results

Questionnaires were delivered to 3000 people, and the number of valid responses with the preferred place of death of the respondent was 1781. Breakdowns are their own home (n = 805, 45.2%), descendants’ home (n = 32, 1.8%), house of relatives such as siblings (n = 3, 0.2%), hospitals (n = 466, 26.2%), elderly housings with supportive services (n = 67, 3.8%), intensive nursing homes for the elderly (n = 69, 3.9%), unknown (n = 285, 15.7%), and others (n = 54, 3.0%).

After excluding data with missing values and answers of “unknown” or “others” for the question of the preferred place of death, a total of 1133 responses were included in the analyses in this study. Neither multicollinearity nor particular outliers were observed. The Hosmer and Lemeshow test showed significant goodness of fit (p = 0.219). A cross table, crude odds ratios, p-value, and adjusted odds ratios are shown in Table 1.

**Table 1:** Cross-table, crude odds ratios, and adjusted odds ratios

Item	Preferred place of death		Crude odds ratio (95% CI)	p-value	Adjusted odds ratio (95% CI)
	Home	Institution			
Age (years)					
65–74	301	194	1.097 (0.707–1.701)	0.703	1.102 (0.670–1.813)
75–84	298	241	0.874 (0.566–1.350)	0.429	0.824 (0.511–1.330)
85+	58	41	Reference		Reference
Gender					
Male	489	285	1.951 (1.514–2.514)	*< 0.001*	*1.932* (*1.405*–*2.657*)
Female	168	191	Reference		Reference
Residential district					
Community general support center East	128	93	0.996 (0.669–1.483)	0.368	0.815 (0.522–1.272)
Community general support center West	90	59	1.104 (0.710–1.717)	0.697	0.908 (0.561–1.472)
Community general support center North	82	44	1.349 (0.843–2.159)	0.869	0.956 (0.563–1.623)
Community general support center Central	133	98	0.982 (0.662–1.457)	0.303	0.803 (0.528–1.219)
Community general support center Tama Center	119	106	0.813 (0.548–1.206)	0.494	0.864 (0.567–1.315)
Community general support center South	105	76	Reference		Reference
Residence					
Detached house	377	212	Reference		Reference
Housing complex of one story	79	60	0.740 (0.509–1.078)	0.250	0.777 (0.505–1.195)
Housing complex of more than two stories without elevators	144	142	0.570 (0.428–0.759)	*0.002*	*0.586* (*0.415*–*0.826*)
Housing complex of more than two stories with elevators	57	62	0.517 (0.348–0.769)	*0.008*	*0.547* (*0.351*–*0.854*)
Residence year					
≥ 20 years	499	347	1.174 (0.896–1.538)	0.959	0.993 (0.743–1.326)
< 20 years	158	129	Reference		Reference
Preference for continued living in the city					
Yes	564	382	1.492 (1.089–2.044)	*0.035*	*1.438* (*1.026*–*2.017*)
No or either way	93	94	Reference		Reference
Family type					
Living alone	78	100	0.568 (0.373–0.864)	0.910	1.028 (0.631–1.676)
Living with only a spouse	355	202	1.280 (0.907–1.805)	*0.024*	*1.519* (*1.058*–*2.180*)
Living with relatives other than a spouse	121	99	0.890 (0.597–1.326)	0.147	1.403 (0.887–2.220)
Living with a spouse and relatives	103	75	Reference		Reference
Health conditions					
Healthy	557	379	1.426 (1.047–1.940)	*0.034*	*1.490* (*1.031*–*2.153*)
Unhealthy	100	97	Reference		Reference
Certified need for long-term care					
None	595	434	Reference		Reference
Requiring support	31	26	0.870 (0.509–1.486)	0.436	1.280 (0.688–2.381)
Requiring long-term care	31	16	1.413 (0.763–2.616)	0.068	1.982 (0.950–4.133)
IADL					
Independent	544	401	Reference		Reference
Decreased functioning	113	75	1.111 (0.807–1.528)	0.713	1.079 (0.720–1.616)
Contribution to community					
Yes	464	306	1.336 (1.038–1.718)	0.511	1.104 (0.822–1.481)
No	193	170	Reference		Reference
Level of social activities (Continuous variable)			0.976 (0.955–0.997)	0.516	0.990 (0.961–1.020)
Affordability					
High	509	376	0.915 (0.687–1.218)	*0.029*	*0.694* (*0.499*–*0.964*)
Low	148	100	Reference		Reference
Communication with family					
Inadequate	68	61	0.785 (0.544–1.135)	0.610	0.900 (0.599–1.351)
Adequate	589	415	Reference		Reference
Communication with friends					
Inadequate	231	186	0.845 (0.662–1.079)	0.286	0.850 (0.631–1.146)
Adequate	426	290	Reference		Reference

Italic cells: significant factors

The adjusted odds ratios and 95% CIs showed that those who prefer dying at home were: (1) male participants; (2) those who lived in detached houses, compared with those who lived in housing complexes of more than two stories (with or without elevators); (3) those with a preference for continued living in the city; (4) those who lived with only a spouse (i.e., no secondary caregiver), compared with those who lived with a spouse and relatives; (5) those in good health; and (6) those with low affordability.

## Discussion

The results revealed that 47% of the participants preferred home death including a death at home of relatives or descendants. The home death rate in 2015 was 12.7%, showing a large gap between patient preference for home death and rate of actual home death.

The multivariable analysis excluding missing data showed that preference for home death was significantly affected by six factors: gender, residence with more than two stories, preference to continue to live in the city, family type, health conditions, and affordability. A higher preference for home death in male participants may be due to various factors such as paternalistic attitude, financial status, relative unsociability, attachment to home, etc. Regarding family type, the results showed that those who lived with only a spouse were more likely to prefer dying at home than those who lived with a spouse and other family members (e.g., relatives). As a way of thinking of this generation, it is inferred that men have a low psychological barrier for having their spouses take care of them but an uncomfortable feeling to be taken care of by other family members. These findings on the two factors were in opposition to those in the abovementioned previous study by Takeu [8]. This may be due to the difference in study design as this study was a survey of residence including healthy individuals while the study by Takeu was a retrospective study of home deaths.

The analysis also revealed other factors that have not been examined. Regarding the type of residence, those who lived in a detached house were more likely to prefer dying at home than those who lived in a housing complex of more than two stories. According to “Results of Survey on the Senior Citizens’ Attitude toward Housing and the Living Environment” for FY 2014 by the Cabinet Office, Government of Japan, in those aged 60 and over, the rates of those who were “satisfied,” “moderately satisfied,” “moderately dissatisfied,” and “dissatisfied” with their detached houses were 33.9, 43.6, 14.9, and 4.3%, respectively. The rates of those who were “satisfied,” “moderately satisfied,” “moderately dissatisfied,” and “dissatisfied” with their housing complexes were 22.5, 48.8, 16.5, and 7.2%, respectively, indicating that those who lived in detached houses were significantly more satisfied with their housing [14]. It is suggested that the tendency to be satisfied with detached houses leads to a preference for home death.

Another factor may be the ease of remodeling the home when a healthy elderly person needs nursing care. The fact that those who lived on the first floor of a housing complex required less support when going out, making little difference from those who lived in detached houses, may have influenced the preference for home death. In Tama City, there is a system that allows the frail elderly living on higher floors (e.g., 4th and 5th floors) of housing complexes without elevators to move to rooms on lower floors when they become vacant. This may also have influenced the preference for home death. It can be inferred that the “preference for continued living in the city” may reflect the fact that they are satisfied with the current situation. Moving to a hospital or other medical facility means moving out of their homes and thus may cause anxiety that they may not be able to continue to live in the city.

Healthy individuals were more likely to prefer dying at home than those who were unhealthy. Those who became ill may tend to think that they will need more personal and nursing care in the near future and that they do not want to put the burden of nursing care on their families. However, their health conditions may change in the future. Thus, such a questionnaire study does not provide sufficient information on how they will feel when they need nursing care in the future.

Those with high affordability were more likely to prefer dying at home than those with low affordability. The higher the affordability, the more flexible nursing care services they can choose. However, affordability of staying in a hospital or other medical facility seemed to be their concern.

### Limitations

This study has several limitations. First, the response rate was low, and many data were excluded from the multivariable analysis due to participant withdrawals, etc. Second, the key question on home death to identify the dependent variable was simple (i.e., “Where do you prefer to die?”) without any context. Some participants might feel difficulty in response to this question.

## Conclusions

This study identified six factors contributing to the preference for home death: (1) male participants; (2) those who lived in detached houses, compared with those who lived in housing complexes of more than two stories (with or without elevators); (3) those with a preference for continued living in the city; (4) those who lived with only a spouse, compared with those who lived with a spouse and relatives; (5) those in good health; and (6) those with low affordability. For the following two factors, the findings were in opposition to those in the abovementioned previous study: “male participants” and “those who lived with only a spouse (i.e., no secondary caregiver).” The other four factors have not been examined before. This may be due to the fact that this study was a resident survey of healthy and frail elderly.

Future studies will include a similar survey in other regions to increase the generalizability of the results. Future studies will also analyze qualitative interview data to examine psychological factors of the dependent variable (i.e., “preferred place of death”).

## References

[1] Ministry of Health, Labor and Welfare. Annual vital statistics report (Final); 2015. http://www.mhlw.go.jp/toukei/saikin/hw/jinkou/kakutei15/. Accessed 10 Feb 2018.

[2] Cabinet Office, Government of Japan. Trends in deaths. http://www8.cao.go.jp/kisei-kaikaku/kaigi/meeting/2013/wg4/kenko/151224/item2-2-2.pdf. Accessed 10 Feb 2018.

[3] Shinya Matsuda. The results of the study by the working group for the analysis and discussion of medical and nursing care information—about the estimated hospital bed capacity by medical care function for 2025. http://www.kantei.go.jp/jp/singi/shakaihoshoukaikaku/chousakai_dai5/siryou1.pdf. Accessed 10 Feb 2018.

[4] Gomes B, Higginson IJ (2006). Factors influencing death at home in terminally ill patients with cancer: systematic review. BMJ.

[5] Wahid AS, Sayma M, Jamshaid S, Kerwat D, Oyewole F, Saleh D (2017). Barriers and facilitators influencing death at home: a meta-ethnography. Palliat Med.

[6] Palliative Care Meeting. About the results of “the Surveys of Palliative Care”; 2010. http://www.mhlw.go.jp/bunya/iryou/zaitaku/dl/07.pdf. Accessed 10 Feb 2018.

[7] The Review Committee on Awareness Surveys of Palliative Care, etc. Awareness survey report on palliative care; 2014. http://www.mhlw.go.jp/bunya/iryou/zaitaku/dl/h260425-02.pdf. Accessed 10 Feb 2018.

[8] Takeu R (2008). A review of articles about factors association with death at home and death at hospital since 1990 in japan. Nihon Chiiki Kango Gakkai Shi.

[9] Hattori A, Uemura K, Masuda Y, Mogi N, Naito M, Iguchi A (2001). Factors contributing to dying at home in elderly patients who received home care service. Nihon Ronen Igakkai Zasshi.

[10] Suzuki H, Suzuki S (2005). Factors for dying at home in home care for terminally-ill cancer patients. Japan J Prim Care.

[11] Sugikoto S, Koga T, Nishigaki C (2009). Problems of home medical treatment in terminal medical care. Bull Soc Med.

[12] Tama City. Elderly health care and public aid project (insured long-term care service plans) for 2015–2017; 2015. http://www.city.tama.lg.jp/0000003472.html. Accessed 10 Feb 2018.

[13] Tama City Medical Association. Research project on multi-sectoral collaboration of medical, nursing and preventive care in comprehensive community care for home care promotion: a report on long-term preventative care and disaster support for the elderly. 2015. p. 1–215.

[14] Cabinet Office, Government of Japan. Survey on the senior citizens’ attitude toward housing and the living environment for FY 2014. http://www8.cao.go.jp/kourei/ishiki/h26/sougou/zentai/index.html. Accessed 10 Feb 2018.

